# Supplementary material for: Interspecific synchrony on breeding performance and the role of anthropogenic food subsidies
Source: PLoS One. 2022 Oct 12;17(10):e0275569. doi: 10.1371/journal.pone.0275569 (PMC9555664; doi:10.1371/journal.pone.0275569)
Supplement: S1 Table — (DOCX) [file pone.0275569.s001.docx]

**Table S1 Study data summary.**

| Year | VLm | SE | VCd | SE | n_Lm_ | n_Cd_ |
| --- | --- | --- | --- | --- | --- | --- |
| 2002 | 76.72 | 1.26 | 72.9 | 0.54 | 60 | 109 |
| 2003 | 74.33 | 1.27 | 73.76 | 0.54 | 66 | 117 |
| 2004 | 75.04 | 1.03 | 72.72 | 0.51 | 105 | 133 |
| 2005 | 74.23 | 0.66 | 75.38 | 0.50 | 174 | 136 |
| 2006 | 73.24 | 0.95 | 73.47 | 0.48 | 135 | 126 |
| 2007 | 74.85 | 0.71 | 72.43 | 0.52 | 183 | 118 |
| 2008 | 74.15 | 0.77 | 71.59 | 0.53 | 150 | 114 |
| 2009 | 77.05 | 0.92 | 72.30 | 0.55 | 153 | 116 |
| 2010 | 73.44 | 0.92 | 74.41 | 0.55 | 99 | 105 |
| 2011 | 71.74 | 1.36 | 73.44 | 0.58 | 45 | 93 |
| 2012 | 68.75 | 1.80 | 70.37 | 0.65 | 27 | 87 |
| 2013 | 72.83 | 1.10 | 72.44 | 0.63 | 60 | 86 |
| 2014 | 72.15 | 1.86 | 71.45 | 0.51 | 51 | 104 |
| 2015 | 71.23 | 1.31 | 70.88 | 0.56 | 78 | 94 |
| 2016 | 72.44 | 0.86 | 72.15 | 0.61 | 168 | 104 |
| 2017 | 75.30 | 1.36 | 72.08 | 0.60 | 57 | 89 |
| 2018 | 71.04 | 0.92 | 72.93 | 0.50 | 81 | 95 |
| 2019 | 72.22 | 1.52 | 70.31 | 0.64 | 51 | 71 |

Mean egg volume and number of eggs (n) by year and species considered for the analysis. Notation: Lm, Yellow-Legged Gull and Cd, Scopoli’s Shearwater.
