# Supplementary material for: Interspecific synchrony on breeding performance and the role of anthropogenic food subsidies
Source: PLoS One. 2022 Oct 12;17(10):e0275569. doi: 10.1371/journal.pone.0275569 (PMC9555664; doi:10.1371/journal.pone.0275569)
Supplement: S2 Table — (DOCX) [file pone.0275569.s002.docx]

**Table S2 Extended model results**

| **Model** | **R^2^** | **df** | **logLik** | **AIC_c_** | **ΔAIC_c_** | **ω** |
| --- | --- | --- | --- | --- | --- | --- |
| Sp + sync + W_NAO_ + Sp:sync | 0.52 | 6 | -58.14 | 131.17 | 0.00 | 0.43 |
| Sp + sync + W_NAO_ + Sp:sync + Sp:wnao | 0.53 | 7 | -57.72 | 133.44 | 2.27 | 0.14 |
| Sp + sync + W_NAO_ | 0.44 | 5 | -60.89 | 133.78 | 2.61 | 0.12 |
| Sp + sync + W_NAO_ + Sp:sync + sync: W_NAO_ | 0.52 | 7 | -58.13 | 134.27 | 3.10 | 0.09 |
| sync + W_NAO_ | 0.38 | 4 | -62.78 | 134.86 | 3.69 | 0.07 |
| Sp + sync + W_NAO_ + Sp:wnao | 0.46 | 6 | -60.34 | 135.58 | 4.41 | 0.05 |
| Sp + sync + W_NAO_ + sync: W_NAO_ | 0.44 | 6 | -60.89 | 136.67 | 5.50 | 0.03 |
| Sp + sync + W_NAO_ + Sp:sync + Sp: W_NAO_ + sync: W_NAO_ | 0.53 | 8 | -57.72 | 136.77 | 5.60 | 0.03 |
| sync + W_NAO_ + sync: W_NAO_ | 0.38 | 5 | -62.78 | 137.56 | 6.39 | 0.02 |
| Sp + sync + W_NAO_ + Sp: W_NAO_ + sync: W_NAO_ | 0.46 | 7 | -60.34 | 138.68 | 7.51 | 0.01 |
| Sp + sync + W_NAO_ + Sp:sync + Sp: W_NAO_ + sync: W_NAO_ + Sp:sync: W_NAO_ | 0.54 | 9 | -57.36 | 139.63 | 8.47 | 0.01 |
| Sp + sync + Sp:sync | 0.33 | 5 | -64.00 | 140.01 | 8.84 | 0.01 |
| Sp + sync | 0.25 | 4 | -66.03 | 141.35 | 10.19 | 0.00 |
| sync | 0.19 | 3 | -67.47 | 141.70 | 10.53 | 0.00 |
| Sp + W_NAO_ | 0.21 | 4 | -66.92 | 143.14 | 11.97 | 0.00 |
| W_NAO_ | 0.15 | 3 | -68.30 | 143.35 | 12.18 | 0.00 |
| Sp + W_NAO_ + Sp: W_NAO_ | 0.23 | 5 | -66.53 | 145.06 | 13.90 | 0.00 |
| 1 | 0.00 | 2 | -71.27 | 146.91 | 15.74 | 0.00 |
| Sp | 0.06 | 3 | -70.11 | 146.98 | 15.81 | 0.00 |
| t | 0.63 | 19 | -53.29 | 192.09 | 60.92 | 0.00 |
| sync + t | 0.63 | 19 | -53.29 | 192.09 | 60.92 | 0.00 |
| W_NAO_ + t | 0.63 | 19 | -53.29 | 192.09 | 60.92 | 0.00 |
| sync + W_NAO_ + t | 0.63 | 19 | -53.29 | 192.09 | 60.92 | 0.00 |
| sync + W_NAO_ + t + sync: W_NAO_ | 0.63 | 19 | -53.29 | 192.09 | 60.92 | 0.00 |
| Sp + t | 0.69 | 20 | -49.96 | 195.91 | 64.74 | 0.00 |
| Sp + sync + t | 0.69 | 20 | -49.96 | 195.91 | 64.74 | 0.00 |
| Sp + W_NAO_ + t | 0.69 | 20 | -49.96 | 195.91 | 64.74 | 0.00 |
| Sp + sync + W_NAO_ + t | 0.69 | 20 | -49.96 | 195.91 | 64.74 | 0.00 |
| Sp + sync + W_NAO_ + t + sync: W_NAO_ | 0.69 | 20 | -49.96 | 195.91 | 64.74 | 0.00 |
| Sp + sync + W_NAO_ + t + Sp:sync | 0.77 | 21 | -44.53 | 197.06 | 65.89 | 0.00 |
| Sp + sync + W_NAO_ + t + Sp:sync + sync: W_NAO_ | 0.77 | 21 | -44.53 | 197.06 | 65.89 | 0.00 |
| Sp + sync + t + Sp:sync | 0.77 | 21 | -44.53 | 197.06 | 65.89 | 0.00 |
| Sp + W_NAO_ + t + Sp: W_NAO_ | 0.71 | 21 | -48.94 | 205.87 | 74.70 | 0.00 |
| Sp + sync + wnao + t + Sp: W_NAO_ | 0.71 | 21 | -48.94 | 205.87 | 74.70 | 0.00 |
| Sp + sync + W_NAO_ + t + Sp: W_NAO_ + sync: W_NAO_ | 0.71 | 21 | -48.94 | 205.87 | 74.70 | 0.00 |
| Sp + sync + W_NAO_ + t + Sp:sync + Sp:wnao | 0.78 | 22 | -43.63 | 209.10 | 77.93 | 0.00 |
| Sp + sync + W_NAO_ + t + Sp:sync + Sp: W_NAO_ + sync: W_NAO_ | 0.78 | 22 | -43.63 | 209.10 | 77.93 | 0.00 |
| Sp + sync + W_NAO_ + t + Sp:sync + Sp: W_NAO_ + sync: W_NAO_ + Sp:sync: W_NAO_ | 0.79 | 23 | -42.83 | 223.66 | 92.49 | 0.00 |

Modelling of egg volume of Yellow-legged Gulls and Scopoli’s Shearwaters on Dragonera Natural Park, Spain. Top competing models within 4 ΔAIC_c_ points are shown in bold. Notations are Sp, species; sync, synchrony; W_NAO_, Winter North Atlantic Oscilation Index; t, year; “+”, additive effect; “:”, interaction effect; df, degrees of freedom; logLik, Log-Likelihood, AICc, Akaike’s information criterion corrected for sample size; ΔAICc, AICc difference with the best model; w, weight of the model.
