# Supplementary material for: Interspecific synchrony on breeding performance and the role of anthropogenic food subsidies
Source: PLoS One. 2022 Oct 12;17(10):e0275569. doi: 10.1371/journal.pone.0275569 (PMC9555664; doi:10.1371/journal.pone.0275569)
Supplement: S3 Table — (DOCX) [file pone.0275569.s003.docx]

**Table S3 Model averaging estimates**

| **Parameter** | **Estimate** | **95% CI** |
| --- | --- | --- |
| i | 73.150 | [72.19, 74.11] |
| **β_Lm_** | **1.457** | **[0.16, 2.75]** |
| β_SyncYES_ | -0.983 | [-2.50, 0.53] |
| **β_WNAO_** | **-0.388** | **[-0.69, -0.08]** |
| **β_Lm_:Sync_YES_** | **-2.014** | **[-3.85, -0.18]** |
| β_Lm:WNAO_ | 0.191 | [-0.26, 0.64] |
| β_SyncYES: WNAO_ | -0.015 | [-0.58, 0.55] |

Model averaging estimates and 95 % confidence intervals from best competing models (ΔAIC_c_<4, as in table 1). Notation: i, intercept; β, beta estimate; Lm: Yellow-legged gull; SyncYES, synchrony period 2010-2016; WNAO, Winter North Atlantic Oscillation Index; “:”, interaction.
