# Supplementary material for: Interspecific synchrony on breeding performance and the role of anthropogenic food subsidies
Source: PLoS One. 2022 Oct 12;17(10):e0275569. doi: 10.1371/journal.pone.0275569 (PMC9555664; doi:10.1371/journal.pone.0275569)
Supplement: S1 Appendix — (DOCX) [file pone.0275569.s004.docx]

**Appendix S4: Code**

######

#### Clean the workspace

rm(list=ls())

###load libraries

if(!require(devtools)) install.packages("devtools")

devtools::install_github("kassambara/ggpubr")

library(ggpubr)

library(ggpmisc)

library(boot)

library(grid)

library(lme4)

library(MuMIn)

library (AICcmodavg)

library(ggplot2)

# Multiple plot function####

#

# ggplot objects can be passed in ..., or to plotlist (as a list of ggplot objects)

# - cols: Number of columns in layout

# - layout: A matrix specifying the layout. If present, 'cols' is ignored.

#

# If the layout is something like matrix(c(1,2,3,3), nrow=2, byrow=TRUE),

# then plot 1 will go in the upper left, 2 will go in the upper right, and

# 3 will go all the way across the bottom.

#

multiplot <- function(..., plotlist=NULL, file, cols=1, layout=NULL) {

# Make a list from the ... arguments and plotlist

plots <- c(list(...), plotlist)

numPlots = length(plots)

# If layout is NULL, then use 'cols' to determine layout

if (is.null(layout)) {

# Make the panel

# ncol: Number of columns of plots

# nrow: Number of rows needed, calculated from # of cols

layout <- matrix(seq(1, cols * ceiling(numPlots/cols)),

ncol = cols, nrow = ceiling(numPlots/cols))

}

if (numPlots==1) {

print(plots[[1]])

} else {

# Set up the page

grid.newpage()

pushViewport(viewport(layout = grid.layout(nrow(layout), ncol(layout))))

# Make each plot, in the correct location

for (i in 1:numPlots) {

# Get the i,j matrix positions of the regions that contain this subplot

matchidx <- as.data.frame(which(layout == i, arr.ind = TRUE))

print(plots[[i]], vp = viewport(layout.pos.row = matchidx$row,

layout.pos.col = matchidx$col))

}

}

}

##################################################

####### REAL DATA #################

##################################################

data<-read.csv("data_syncrhony.csv",sep=";")

data

Lm<-data[which(data$SP=="LM"),3]

Cd<-data[which(data$SP=="CD"),3]

X<-cbind(Lm,Cd)

X<-t(X)

##################################################

####### SIMULATED DATA #################

##################################################

### we randomize the data for each species. We resample our data with no replacement.

###

niter<-1000

Xs<-array(0,dim=c(dim(X)[1],dim(X)[2],niter))

for (k in 1:niter){#loop on iterations

for( i in 1:dim(X)[1]){# loop on species

Xs[i,,k]<-sample(X[i,], replace = FALSE)

}}

#############################

###### Function to get Y ####

#############################

DataTrans<-function(X){

n<-dim(X)[1]

year<-dim(X)[2]

moccasions<-year-2

m<-array(, dim=c(3,2*moccasions,n))

for (s in 1:n){# loop for SpecieS

r<-X[s,]

for (j in 1:moccasions){# loop for time

for (i in 1:3){# loop for rowS

position<-i+j-1

m[i,2*j-1,s]<-r[position]

}#rowS

}#time

}#SpecieS

m

###loop to find maximum and minimum of the three valueS. Maximum value iS Set to 2, minimum to 0

moccasions

for(s in 1:n){

for(j in 1:(moccasions)){#loop on time

which.max( m[,2*j-1,s])->max

which.min( m[,2*j-1,s])->min

m[max,2*j,s]<-2

m[min,2*j,s]<-0

}#time

}#SpecieS

# na correSpondS to the other value not previouSly claSified we replace it by 1.

m[is.na(m)] <- 1

m

##loop to create a new vector containing the diferent combinationS.

g<-matrix(NA, nrow = n, ncol = moccasions)

for (s in 1:n){#loop SpecieS

for (j in 1:(moccasions)){

a<-m[1,2*j,s]

b<-m[2,2*j,s]

c<-m[3,2*j,s]

g[s,j]<-100*a+10*b+1*c

}#loop time

}#loop SpecieS

g<-replace(g,which(g==201),1)

g<-replace(g,which(g==12),3)

g<-replace(g,which(g==21),2)

g<-replace(g,which(g==102),1)

g<-replace(g,which(g==120),2)

g<-replace(g,which(g==210),4)

data<-g

return(data)

}

#################

###### GET Y ###

#################

#From real data

Yr<-DataTrans(X)

#From simulated data

Ys<-array(0,dim=c(dim(Xs)[1],dim(Xs)[2]-2,dim(Xs)[3]))

for (j in 1:dim(Xs)[3]){

Ys[,,j]<-DataTrans(Xs[,,j])}

###################################

###################################

###### GET p FUNCTION #######

###################################

###################################

##### P for all species together

getP<-function(X){

### we are going to calculate p which iS the obServed proportion of occaSionS where the

### SpecieS egg volume/clutch SiXe increaSeS between two time-pointS:

year<-dim(X)[2]-1

sp<-dim(X)[1]

Y<-matrix(0, nrow = sp, ncol = year)

Y

for (s in 1:sp){

for(j in 1:year){

if (X[s,j]<=X[s,j+1]){

Y[s,j]<-1} else Y[s,j]<-0

}#time

}#SpecieS

delta<-sum(Y)

# the probability iS obtained with the following formula

#p=1/n*(X-1)* delta ## delta iS the number of timeS Xt<=Xt+1

p<-(1/(sp*year))*delta

p

return(p)}

## this p is calculated for all species!! I might need to do it for each one of them

##### P for each species

getPspecies<-function(X){

### we are going to calculate p which iS the obServed proportion of occaSionS where the

### SpecieS egg volume/clutch SiXe increaSeS between two time-pointS:

year<-dim(X)[2]-1

sp<-dim(X)[1]

Y<-matrix(0, nrow = sp, ncol = year)

Y

for (s in 1:sp){

for(j in 1:year){

if (X[s,j]<=X[s,j+1]){

Y[s,j]<-1} else Y[s,j]<-0

}#time

}#SpecieS

delta<-rowSums(Y)

# the probability iS obtained with the following formula

#p=1/n*(X-1)* delta ## delta iS the number of timeS Xt<=Xt+1

p<-(1/(year))*delta

p

return(p)}

#################################

#################################

#################################

#p from real data

Yrp<-getP(X)

Yrpsp<-getPspecies(X)

#p from simulated data

Ysp<-array(0,dim(Xs)[3])

for (j in 1:dim(Xs)[3]){

Ysp[j]<-getP(Xs[,,j])}

Yspsp<-array(0,dim=c(dim(Xs)[3],dim(Xs)[1]))

for (j in 1:dim(Xs)[3]){

Yspsp[j,]<-getPspecies(Xs[,,j])}

#################################

#################################

###### ##########

###### Entropy ##########

###### ##########

#################################

#################################

##function definition##

enthropy<-function(D){

moccasions<-dim(D)[2]

states<-4

##PROPORTION OF SPECIES IN EACH STATE

S<-matrix(,ncol=moccasions,nrow=states)

for (j in 1:moccasions){

M<-D[,j]

c1<-0

c2<-0

c3<-0

c4<-0

for (i in 1:dim(D)[1]){

if ( M[i]==1 ) c1<-c1+1 else c1<-c1

if ( M[i]==2 ) c2<-c2+1 else c2<-c2

if ( M[i]==3 ) c3<-c3+1 else c3<-c3

if ( M[i]==4 ) c4<-c4+1 else c4<-c4

}

S[1,j]<-c1

S[2,j]<-c2

S[3,j]<-c3

S[4,j]<-c4}

S<-S/dim(D)[1]

##OBSERVED ENTROPY ACROSS THE COMMUNITY

H<-matrix(,ncol=moccasions,nrow=1)

PRO<-matrix(,ncol=moccasions,nrow=states)

for(j in 1:moccasions){

for(e in 1:states){

PRO[e,j]<--S[e,j]*log(S[e,j])# if there iS no SpecieS in a State the logarithm iS undefined

}}

PRO[is.na(PRO)] <- 0

PRO

for(j in 1:moccasions){

H[j]<-colSums(PRO)[j]

}

list(H,S)}

##end entropy function

graph<- enthropy(Yr)

HH<-graph[1]

SS<-graph[2]

Ht <- matrix(unlist(HH), ncol=dim(Yr)[2])

S <- matrix(unlist(SS), ncol=dim(Yr)[2])

Hnull<-matrix(0,nrow=niter,ncol=dim(Ys)[2])

for (j in 1:niter){

graph<- enthropy(Ys[,,j])

H<-graph[1]

Hnull[j,] <- matrix(unlist(H), ncol=dim(Ys)[2])

}

meanHnull<-matrix(0,ncol=5,nrow=dim(Hnull)[2])

for (i in 1:dim(Hnull)[2]){

meanHnull[i,1]<-mean(Hnull[,i])

meanHnull[i,2]<-sd(Hnull[,i])

meanHnull[i,3]<-meanHnull[i,2]/sqrt(niter)

meanHnull[i,4]<-meanHnull[i,1]+1.96*meanHnull[i,3]

meanHnull[i,5]<-meanHnull[i,1]-1.96*meanHnull[i,3]

colnames(meanHnull) <- c("MEAN","SD","SD/N","UPP","LOW")

}

#########################################

#########################################

####### #########

####### Synchrony value #########

####### #########

#########################################

#########################################

phi<-matrix(0,nrow=dim(meanHnull)[1],ncol=1)

for (i in 1:dim(meanHnull)[1]){

phi[i,]<-1-(Ht[1,i]/meanHnull[i,1])

}

library(ggplot2)

#######################################

#######################################

###Plot real data

#######################################

#######################################

p1<-ggplot(data, aes(x=YEAR, y=Mean, group=SP, color=SP)) +

geom_line(size =1) +

geom_point(aes(size=3,alpha=0.8))+

scale_y_continuous(breaks=seq(68,79,by=1),limits=c(68,79),expand=c(0,0))+

scale_colour_manual(values=c(CD="black",LM="grey"))+

geom_errorbar(aes(ymin=Mean-1.96*std, ymax=Mean+1.96*std), width=.3,

position=position_dodge(0.05),size =0.8)+

geom_vline(xintercept = 2009.9, linetype="dotted",

color = "black", size=0.5)+

scale_x_continuous(breaks=seq(2002, 2019, 1))+

labs(title = "",face="bold", x = "Year",

y = expression(paste("Vol ","(mm"^"3",")",sep=""))) +

theme(

# LABLES APPEARANCE

plot.title = element_text(size=14, face= "bold", colour= "black" ),

axis.title.y = element_text(size=16, face="bold", colour = "black"),

axis.text.y = element_text(size=14, colour = "black"), # bold

strip.text.y = element_text(size = 10, face="bold", colour = "black"),

axis.line.y = element_line(colour="black"),

axis.title.x = element_blank(),

axis.text.x = element_blank(),

strip.text.x = element_blank(),

axis.ticks.x = element_blank(),

panel.border = element_blank(),

panel.background = element_blank(),

legend.position = "none")+

scale_size(guide=FALSE)+

scale_alpha(guide=FALSE)+

#annotation_raster(birds, 2017.5, 2019, 76.5, 79)+

annotate("point", x = 2017.5, y = 78, colour = "grey",alpha=0.8,size=4) +

annotate("point", x = 2017.5, y = 77, colour = "black",alpha=0.8,size=4)

#bottom, left, top, and right

par(mar=c(0,5,3,5))

#######################################

###Plot states

#######################################

y.mat <- S

par(mar=c(0,5,0,5))

par(mar=c(0,5,3,5))

par(mar=c())

data_long<-matrix( as.numeric(y.mat),nrow=64)

colnames(data_long)<-"value"

data_long<-as.data.frame(data_long)

data_long$state<-rep(c(1:4),16)

data_long$Year<-rep(c( 2003:2018),each=4)

data_long$state<-as.character(data_long$state)

# Stacked

p2<-ggplot(data_long, aes(fill=state, y=value, x=Year)) +

geom_bar(position="stack", stat="identity", colour="grey") +

scale_fill_manual(values = c("black", "#CCCCCC","darkgrey","white")) +

scale_y_continuous(position = "right",breaks=seq(0,1,by=0.25),limits=c(0,1),expand=c(0,0))+

ylab( expression("% of species in each state ")) +

scale_x_continuous(breaks = seq(2002, 2017, by = 1))+

theme(

# LABLES APPEARANCE

plot.title = element_text(size=14, face= "bold", colour= "black" ),

axis.title.x = element_text(size=14, face="bold", colour = "white"),

axis.title.y = element_text(size=14, face="bold", colour = "black"),

axis.ticks.x = element_blank(), ## <- this line

axis.line.y = element_line(colour="black"),

axis.text.x = element_text(size=10,colour = "white", hjust = 1),

axis.text.y = element_text(size=10, colour = "black"), # bold

strip.text.x = element_text(size = 10, face="bold", colour = "white" ),

strip.text.y = element_text(size = 10, face="bold", colour = "black"),

panel.border = element_blank(),

panel.background = element_blank(),

legend.position = "none",

legend.box = "horizontal",

legend.key=element_blank())+

scale_size(guide=FALSE)+

scale_alpha(guide=FALSE)

#######################################

###Plot synchrony

#######################################

dataH<-as.data.frame(meanHnull)

dataH$Ht<-as.numeric(Ht)

dataH$year<-c(2002:2017)

dataH$phi<-phi

par(mar=c(0,5,3,0))

p3<-ggplot(dataH, aes(year,phi)) +

geom_point(aes())+

geom_line(aes())+

scale_y_continuous(position = "right",breaks=seq(-0.5,1,by=0.25),limits=c(-0.5,1),expand=c(0,0))+

theme(

# LABLES APPEARANCE

plot.title = element_text(size=14, face= "bold", colour= "black" ),

axis.title.x = element_blank(),

axis.title.y = element_blank(),

axis.text.x = element_blank(),

axis.text.y = element_text(size=14, colour = "black"), # bold

strip.text.x = element_blank(),

strip.text.y = element_text(size = 14, face="bold", colour = "black"),

panel.border = element_blank(),

panel.background = element_blank(),

axis.ticks.x = element_blank(), ## <- this line

axis.line.y = element_line(colour="black"),

legend.position = "none",

legend.box = "horizontal",

legend.key=element_blank())+

scale_size(guide=FALSE)+

scale_alpha(guide=FALSE)

multiplot(p1,p2,p3,cols=1)

#################################################

######### Environmental buffering ###############

#################################################

wnao<- c(0.76, 0.2,-0.07,0.12,-1.09,2.79, 2.1,-0.41,-4.64,-1.57,3.17,-1.97,3.1,3.56,0.98,1.47, 0.3,2.09)

year<-c(2002:2019)

data$wnao<-rep(wnao,2)

#get linear model results in each graph

lm_eqn <- function(Cd){

m <- lm(Mean ~ wnao, Cd);

eq <- substitute(italic(y) == a + b %.% italic(x)*","~~italic(r)^2~"="~r2,

list(a = format(coef(m)[1], digits = 2),

b = format(coef(m)[2], digits = 2),

r2 = format(summary(m)$r.squared, digits = 3)))

as.character(as.expression(eq));

}

Lm<-as.data.frame(Lm)

Lm$YEAR<-as.numeric(year)

pre<-subset(Lm,YEAR<2010)

post<-subset(Lm,YEAR>2009)

data$when<-"pre"

data$synchrony<-"no"

data[ which(data$YEAR>2009 & data$YEAR<2017 ),7]<-"yes"

data[ which(data$YEAR>2009),6]<-"post"

Lm<-as.data.frame(data[which(data$SP=="LM"),])

Cd<-as.data.frame(data[which(data$SP=="CD"),])

library(ggpmisc)

par(oma=c(5,5,5,5))

ggplot(data, aes(wnao,Mean)) +

geom_point(aes(size=1.5,alpha=0.8,colour = SP))+

geom_errorbar(aes(ymin=Mean-1.96*std , ymax=Mean+1.96*std ,color=SP ), width=.2)+

geom_smooth(method='lm', col="black")+

scale_colour_manual(values=c("black","gray"))+ ylim(65,80)+

scale_x_continuous(breaks=seq(-5, 5, 1))+

labs(title = "",face="bold", x = expression(paste("W"["NAO"])),

y = expression(paste("Vol","(mm"^"3",")",sep=""))) +

theme(

# LABLES APPEARANCE

plot.title = element_text(size=14, face= "bold", colour= "black" ),

axis.title.x = element_text(size=14, face="bold", colour = "black"),

axis.title.y = element_text(size=14, face="bold", colour = "black"),

axis.text.x = element_text(size=14, colour = "black", hjust = 1),

axis.text.y = element_text(size=14, colour = "black"), # bold

strip.text.x = element_text(size = 14, face="bold", colour = "black" ),

strip.text.y = element_text(size = 14, face="bold", colour = "black"),

panel.border = element_rect(colour = "black", fill=NA, size=0.3),

panel.background = element_rect(fill = NA, colour = "grey50"),

legend.position = "right",

legend.box = "horizontal",

legend.key=element_blank())+

scale_size(guide=FALSE)+

scale_alpha(guide=FALSE)

############# Model results #####################

data$YEAR<-as.factor(data$YEAR)

m1 = glm(data=data,Mean~1+SP+wnao+synchrony+YEAR+SP*wnao*synchrony,family = gaussian(),na.action=na.fail)

library(MuMIn)

dd = dredge(m1)

get.models(dd, subset = 38)[[1]]

ms2 <-subset(dd, delta < 4)

confset.95p <- get.models(dd, cumsum(weight) <= .95)

avgmod.95p <- model.avg(confset.95p) # get averaged coefficients

confint(avgmod.95p)

#or as a 95% confidence set:

aa<-model.avg(object = dd, subset = delta < 4)

m2 = glm(data=data,Mean~1+SP+when++SP*when,family = gaussian(),na.action=na.fail)

summary(m2)
