# Supplementary material for: Interspecific synchrony on breeding performance and the role of anthropogenic food subsidies
Source: PLoS One. 2022 Oct 12;17(10):e0275569. doi: 10.1371/journal.pone.0275569 (PMC9555664; doi:10.1371/journal.pone.0275569)
Supplement: Attachment — Submitted filename: PONE-D-22-11852_R1_Review.docx [file pone.0275569.s006.docx]

I would like to congratulate the authors for their hard work on improving this manuscript and addressing the reviewer’s comments. Most of my concerns were address by simply clarifying the method which was greatly appreciated. The paper has improved significantly, and I only have minor suggestions.

L44: add ‘a’ before pronounced.

L50-51: Sentence is unclear. Suggestion*: Intra-specific synchrony is apparent a at large spatial scale from correlated environmental conditions, the so-called ‘Moran’s effect’*. I’m not sure if this is what you were trying to say.

L63-65: I’m having trouble connecting the start and end of this sentence. I suggest making it into 2 sentences: *Breeding parameter – such as egg volume- often reflect conditions in the ecosystems. Therefore, using inter-specific synchrony of breeding parameters could be a useful indicator of local environment state.*

Line 67: Your discussion now clearly answers your hypothesis, but I would mention your subsidy-decoupling hypothesis somewhere in your discussion.

Line 83-84: I don’t think a subsection is necessary here.

Line 93-123: This paragraph is very long. I suggest merging line 93-96 to previous paragraph and splitting the paragraph by species for the remaining lines (splitting somewhere around 108).

Line 108: Does YLG always breed close by to shearwaters or is that inly in the Mallorca region?

Line 121-123: I appreciate the addition; however, I’m still craving for more detail. Suggestion: add ‘*so that egg volume is higher when environmental stochasticity is removed’* at the end of the sentence.

Line 137-138: Thank you for clarifying that you calculated the mean of egg volume per clutch prior to calculating the annual mean. Maybe mean egg volume per clutch instead? Was the annual mean used for both the synchrony and the GLM? At line 190-191, you only refer to ‘mean egg volume’ which suggest to me that you used mean egg volume per clutch and not the annual mean (which is appropriate for the GLM). If so, the annual mean should only be mentioned in the synchrony part. If not correct line 190-191.

Line 204: Add a sentence precising that synchrony stopped after 2017. This is a big part of the discussion but was not apparent to me until then.

Line 222: Correlation? Why not the actual relationship with you B estimate? Do you have an actual value of R2 to add to the figure?

Line 228-252: Long paragraph and a little confusing. I would first split at line 236. Then I would reshuffle the sentences around: all sentence mentioning overlap first, then YLG foraging strategies. I can see where you are going with this, it just lacks a clear flow.

Line 229: add ‘s’ to ‘regulation’

Line 234: Not sure if I mentioned it in previous revision, I personally don’t think figures should be mentioned in a discussion if they are properly interpreted in the results.

Line 240: ‘…could be the result of changes in foraging strategies and behaviour…’

Line 245: Suggestion: *Although evidence of increase diet overlap between the studied species is lacking…*

Line 253: A second explanation for what?

Line 253-259: You described the drivers of breeding success for shearwaters but not for gulls. What would be the difference between shearwater and gulls that could explain the synchrony? or environmental buffering (see comment line 253).

Line 262-263: Say what was the change in population size (increase or decrease).

Line 265-268: I suggest splitting and rewriting the sentence like this: *However, we argue that the mechanisms from transient dynamic do not explain the disappearance of synchrony after seven years, and that changes in interspecific competition at sea is likely the cause of this pattern.*

Typos and double spaces: Lines 231; 238; 240; 246; 254; 261; 262; 266

Figure 2: Add the value of R2. Y-axis put ‘Annual mean egg volume per clutch’

I’m curious whether you tested the leverage of the data at WNao -5. I’m concerned that it’s driving the slope of your relationship, and it wouldn’t be significant otherwise.
